# Mapping of apparent susceptibility yields promising diagnostic separation of progressive supranuclear palsy from other causes of parkinsonism

**DOI:** 10.1038/s41598-019-42565-4

**Published:** 2019-04-15

**Authors:** Henrik Sjöström, Yulia Surova, Markus Nilsson, Tobias Granberg, Eric Westman, Danielle van Westen, Per Svenningsson, Oskar Hansson

**Affiliations:** 1 Department of Clinical Neuroscience, Karolinska Institutet, Stockholm, 171 65 Sweden; 2 Department of Neurology, Karolinska University Hospital, Stockholm, 141 86 Sweden; 3 Clinical Memory Research Unit, Department of Clinical Sciences, Lund University, 212 24 Malmö, Sweden; 4 Neurology Clinic, Skåne University Hospital, Lund, 221 85 Sweden; 5 Clinical Sciences Lund, Department of Radiology, Lund University, Lund, 221 85 Sweden; 6 Department of Radiology, Karolinska University Hospital, Stockholm, 141 86 Sweden; 7 Division of Clinical Geriatrics, Department of Neurobiology, Care Sciences and Society, Karolinska Institutet, Stockholm, 141 57 Sweden; 8 Department of Neuroimaging, Centre for Neuroimaging Sciences, Institute of Psychiatry, Psychology and Neuroscience, King’s College London, London, SE5 8AF United Kingdom; 9 Diagnostic Radiology, Department of Clinical Sciences, Lund University, Lund, 221 85 Sweden; 10 Department for Image and Function, Skåne University Hospital, Lund, 221 85 Sweden; 11 Memory Clinic, Skåne University Hospital, Malmö, 212 24 Sweden

## Abstract

There is a need for methods that distinguish Parkinson’s disease (PD) from progressive supranuclear palsy (PSP) and multiple system atrophy (MSA), which have similar characteristics in the early stages of the disease. In this prospective study, we evaluate mapping of apparent susceptibility based on susceptibility weighted imaging (SWI) for differential diagnosis. We included 134 patients with PD, 11 with PSP, 10 with MSA and 44 healthy controls. SWI data were processed into maps of apparent susceptibility. In PSP, apparent susceptibility was increased in the red nucleus compared to all other groups, and in globus pallidus, putamen, substantia nigra and the dentate nucleus compared to PD and controls. In MSA, putaminal susceptibility was increased compared to PD and controls. Including all studied regions and using discriminant analysis between PSP and PD, 100% sensitivity and 97% specificity was achieved, and 91% sensitivity and 90% specificity in separating PSP from MSA. Correlations between putaminal susceptibility and disease severity in PD could warrant further research into using susceptibility mapping for monitoring disease progression and in clinical trials. Our study indicates that susceptibility in deep nuclei could play a role in the diagnosis of atypical parkinsonism, especially in PSP.

## Introduction

The most common cause of parkinsonism is idiopathic Parkinson’s disease (PD), and it must be differentiated from progressive supranuclear palsy (PSP) and multiple system atrophy (MSA)^1^. A correct and early diagnosis is important for optimal patient care and for enrolment in clinical trials^2^. However, the parkinsonian disorders may be difficult to distinguish from each other, especially in the early stages. Patients with MSA typically develop autonomic dysfunction in combination with parkinsonian or cerebellar symptoms^3^, whereas patients with PSP commonly exhibit vertical gaze palsy in addition to postural instability and falls^4^. In suspected parkinsonian disorders, magnetic resonance imaging (MRI) is commonly performed as part of the clinical investigation. Structural imaging, however, lacks accuracy in separating these conditions at early stages^5^. Other MRI techniques including diffusion weighted imaging, presence of swallow-tail sign on susceptibility-weighted images (SWI) and neuromelanin-sensitive imaging show varying potential^6–10^.

Quantitative susceptibility mapping (QSM) is a technique where phase MRI data is processed to yield susceptibility maps. The quantitative maps correlate well to tissue iron content^11^. Iron accumulation is common in neurodegenerative disorders, and QSM has been used to investigate changes in the substantia nigra in PD^12,13^. While QSM is commonly gathered through dedicated data acquisition, SWI images acquired using the routine clinical sequence may be used to create maps of, in this case, apparent susceptibility. We have previously shown that PSP and MSA exhibit characteristic susceptibility patterns in a retrospective pilot study using such data^14^. The aim of this study was to corroborate and extend these findings in a larger prospective cohort, investigate a new brain region and to further evaluate the diagnostic performance.

## Results

### Demographics

The demographics of the participants are presented in Table 1. There was no significant difference in age (F_3,195_ = 1.842, p = 0.141 assessed by one-way ANOVA) between the groups. There was a difference in gender distribution (p = 0.026 assessed by Pearson’s χ^2^ test) across all groups, thus a one-way ANCOVA was used to correct for gender and age in the group comparisons. There was no significant difference in gender distribution between the three patient groups (p = 0.170 assessed by Pearson’s χ^2^ test).

**Table 1:** Demographic characteristics of study participants. Abbreviations: IQR = interquartile range; MMSE = mini-mental state examination; MSA = multiple system atrophy; PD = Parkinson’s disease; PSP = progressive supranuclear palsy; UPDRS-III = unified Parkinson’s disease rating scale part III.

Demographic variables	PD	PSP	MSA	Controls
Participants, N	134	11	10	44
Age at clinical visit, y, mean ± SD	66.9 ± 9.6	72.2 ± 5.5	63.4 ± 11.4	66.0 ± 7.8
Gender, F/M; Female, %	48/86; 35	6/5; 55	6/4; 60	26/18; 59
Disease duration, y, mean ± SD	6.0 ± 5.0	5.5 ± 2.8	4.7 ± 2.2	N/A
UPDRS-III, score, median (IQR)	13.5 (7–22.25)	36 (28–58)	38.5 (24–52.5)	1 (0–2)
Hoehn & Yahr, score, median (IQR)	2 (1–2.5)	4 (3–5)	4 (3–5)	0 (0–0)
MMSE, score, median (IQR)	28 (27–29)	27 (19–28)	29 (26.75–29)	29 (28–30)
Dementia, N	18	4	0	0

### Group differences in apparent susceptibility

**Figure 1:**
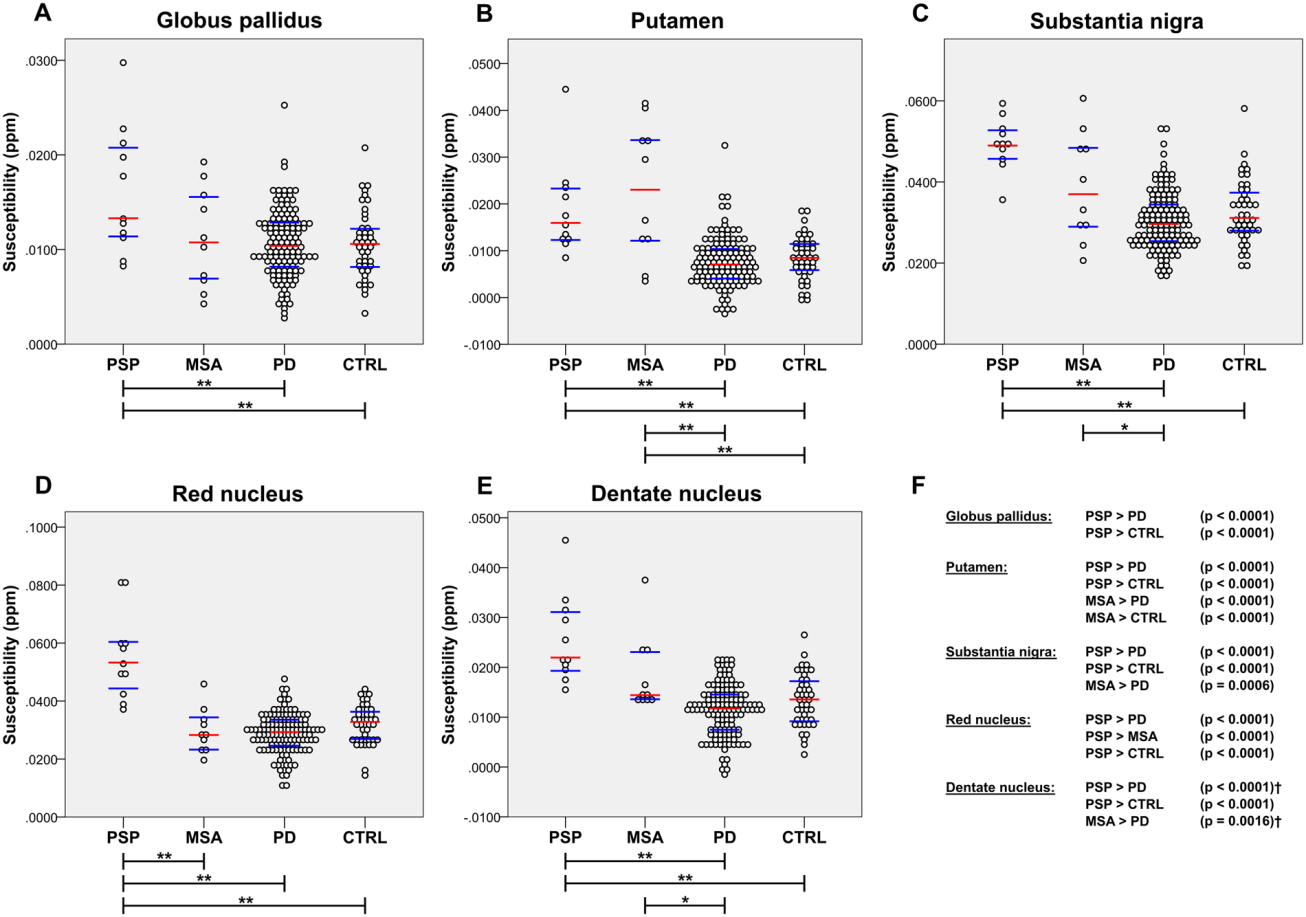
Susceptibility distributions in the different groups in globus pallidus (**A**), putamen (**B**), substantia nigra (**C**), the red nucleus (**D**) and the dentate nucleus (**E**). Red line denotes the median and blue lines the interquartile range. Significant differences between the groups (**F**). Asterisks indicate statistically significant differences between groups: * indicates a p-value less than the Bonferroni-corrected significance level of 0.0017; ** indicates a p-value < 0.0001. Dagger (†) indicates the use of Mann-Whitney U test. Abbreviations: CTRL = control; MSA = multiple system atrophy; PD = Parkinson’s disease; ppm = parts per million; PSP = progressive supranuclear palsy.

**Figure 2:**
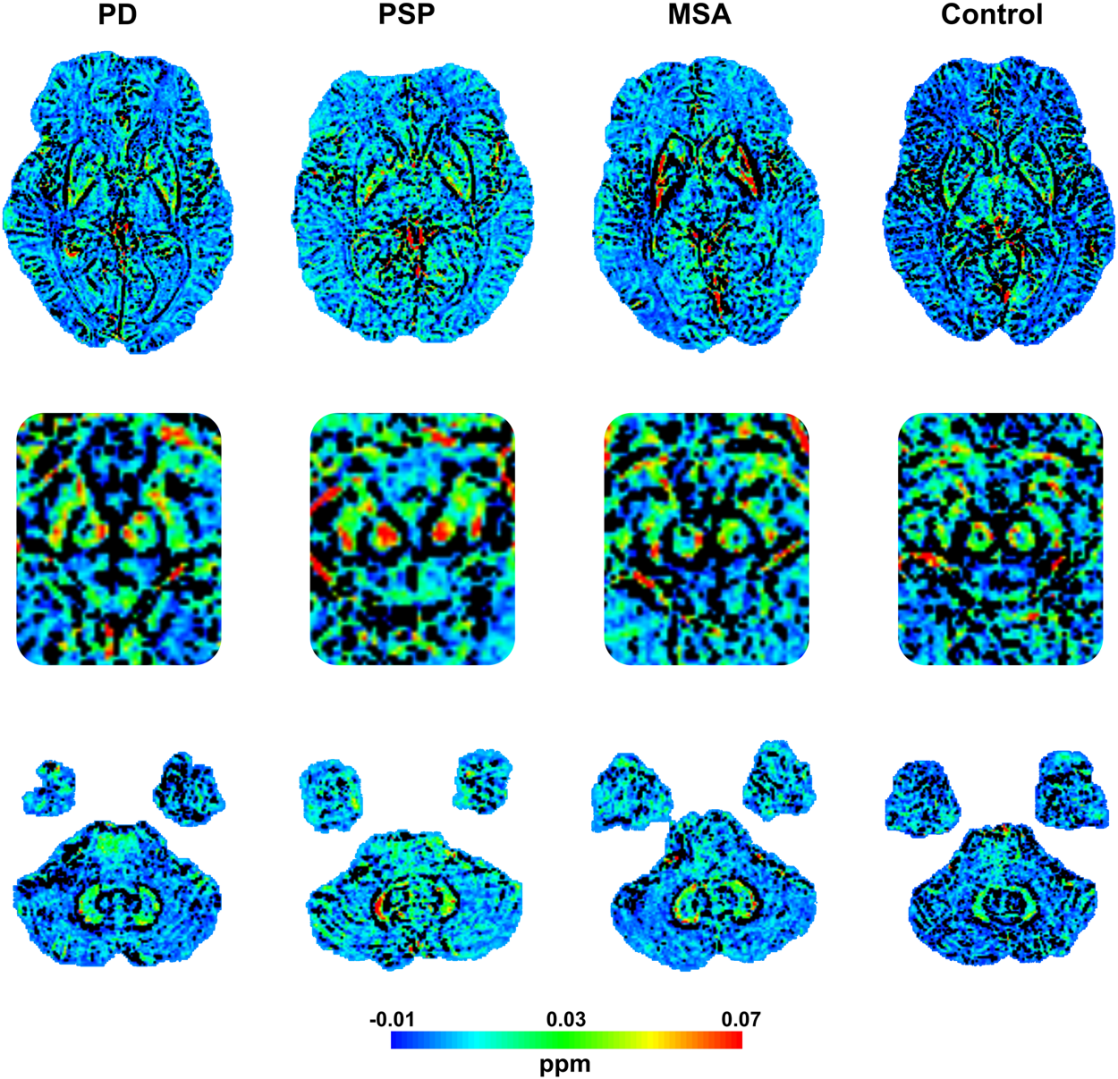
Representative susceptibility maps from the different groups showing higher susceptibility in the red nucleus in PSP, higher susceptibility in the dentate nucleus in PSP and MSA, and higher putaminal susceptibility in MSA compared to PD and controls. Top row showing the level of the lentiform nuclei, middle row mesencephalon and bottom row cerebellum. Abbreviations: MSA = multiple system atrophy; PD = Parkinson’s disease; ppm = parts per million; PSP = progressive supranuclear palsy.

As shown in Fig. 1 there were significant group differences in all regions of interest (ROI); globus pallidus (F_3,193_ = 6.474, p < 0.001), putamen (F_3,193_ = 32.920, p < 0.001), substantia nigra (F_3,193_ = 22.968, p < 0.001), red nucleus (F_3,193_ = 43.137, p < 0.001) and dentate nucleus (F_3,193_ = 26.291, p < 0.001). In the whole cohort susceptibility increased with age in the putamen (p < 0.001), red nucleus (p < 0.001) and dentate nucleus (p = 0.004). No significant gender effects were found in any of the regions. Further analyses revealed that the PSP group showed higher susceptibility in the red nucleus compared to all other groups (all p-values < 0.0001) and in globus pallidus, putamen, substantia nigra and the dentate nucleus compared to PD and controls (all p-values < 0.0001). We also found higher putaminal susceptibility in MSA compared to PD (p < 0.0001) and healthy controls (p < 0.0001), and higher susceptibility in substantia nigra (p = 0.0006) and the dentate nucleus (p < 0.0016) compared to PD. There were no significant differences between the PD group and the control group in any of the ROI. Representative susceptibility maps of the different groups are presented in Fig. 2.

### Diagnostic performance of apparent susceptibility

**Figure 3:**
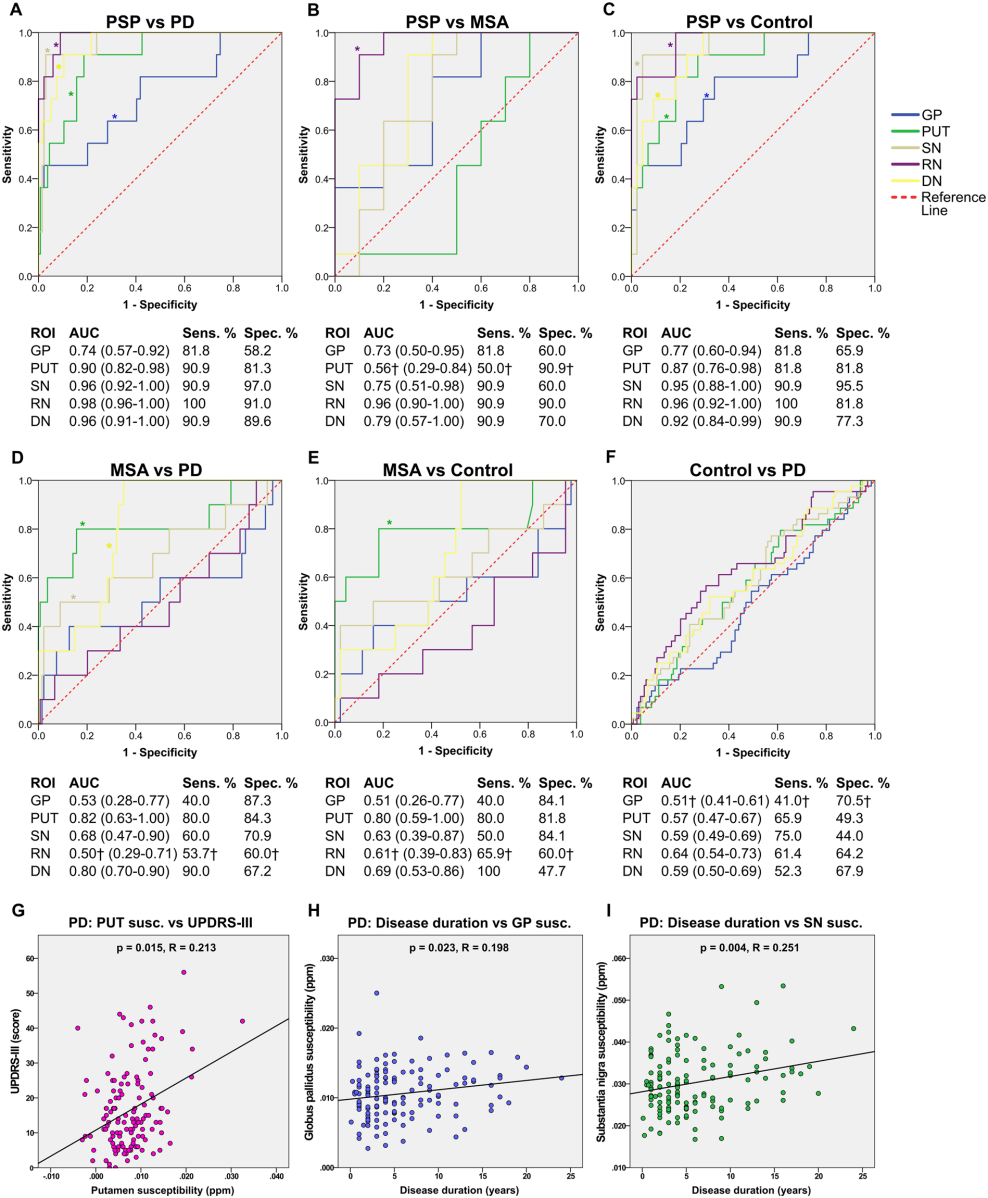
Receiver operating characteristic (ROC) curves displaying diagnostic separation in all regions between PSP and PD (**A**), PSP and MSA (**B**), PSP and controls (**C**), MSA and PD (**D**), MSA and controls (**E**), controls and PD (**F**). Scatter plots showing significant correlations from the Pearson partial correlation tests (**G**–**I**). Curves corresponding to regions with significant differences in the group comparisons are denoted by asterisks (*). Dagger (†) indicates that the AUC, sensitivity and specificity shown are for the reversed comparison. AUC values are presented together with 95% confidence intervals. Abbreviations: AUC = area under curve; DN = dentate nucleus; GP = globus pallidus; MSA = multiple system atrophy; PD = Parkinson’s disease; ppm = parts per million; PSP = progressive supranuclear palsy; PUT = putamen; RN = red nucleus; ROI = region of interest; Sens. = sensitivity; SN = substantia nigra; Spec. = specificity; susc. = susceptibility; UPDRS-III = unified Parkinson’s disease rating scale part III.

Diagnostic performance in different regions between the groups was evaluated using the area under receiver operating characteristic (ROC AUC). The ROC curves and associated results are reported in Fig. 3A–F. Positive and negative predictive values are shown in Table 2. To further investigate the diagnostic accuracy, we performed discriminant analysis between the four groups, using all of the five ROI. In classifying PSP vs. PD, we found a sensitivity of 100%, a specificity of 97.0% and a total of 97.2% cases correctly classified. With leave-one-out cross-validation (LOOCV), results were unchanged. When classifying PSP vs. MSA, a sensitivity of 90.9% and a specificity of 90.0% were achieved, with 90.5% of cases correctly classified. In the LOOCV of this classification we found a sensitivity of 81.8%, a specificity of 90.0% and a total of 85.7% cases correctly classified. Results from the discriminant analyses are reported in Table 3.

**Table 2:** Positive and negative predictive values related to the diagnostic performance shown in Fig. 3. Abbreviations: DN = dentate nucleus; GP = globus pallidus; MSA = multiple system atrophy; NPV = negative predictive value; PD = Parkinson’s disease; PPV = positive predictive value; PSP = progressive supranuclear palsy; PUT = putamen; RN = red nucleus; SN = substantia nigra.

	GP		PUT		SN		RN		DN	
	PPV	NPV	PPV	NPV	PPV	NPV	PPV	NPV	PPV	NPV
PSP vs PD	13.8%	97.5%	28.6%	99.1%	71.4%	99.2%	47.8%	100%	41.7%	99.2%
PSP vs MSA	69.2%	75.0%	66.7%	83.3%	71.4%	85.7%	90.9%	90.0%	76.9%	87.5%
PSP vs Control	37.5%	93.5%	52.9%	94.7%	83.3%	97.7%	57.9%	100%	50.0%	97.1%
MSA vs PD	19.0%	95.1%	27.6%	98.3%	13.3%	96.0%	8.8%	94.7%	17.0%	98.9%
MSA vs Control	36.4%	86.0%	50.0%	94.7%	41.7%	88.1%	28.6%	87.9%	30.3%	100%
Control vs PD	28.2%	80.9%	29.9%	81.5%	30.6%	84.3%	36.0%	83.5%	34.8%	81.3%

**Table 3:** Results from discriminant analyses. Abbreviations: LOOCV = leave-one-out cross-validation; MSA = multiple system atrophy; NPV = negative predictive value; PD = Parkinson’s disease; PPV = positive predictive value; PSP = progressive supranuclear palsy.

	Sensitivity	Specificity	Correctly classified	PPV	NPV	LOOCV Sensitivity	LOOCV Specificity	LOOCV Correctly classified	LOOCV PPV	LOOCV NPV
PSP vs. PD	100%	97.0%	97.2%	73.3%	100%	100%	97.0%	97.2%	73.3%	100%
PSP vs. MSA	90.9%	90.0%	90.5%	90.9%	90.0%	81.8%	90.0%	85.7%	90.0%	81.8%
MSA vs. PD	70.0%	94.8%	93.1%	50.0%	97.7%	60.0%	94.8%	92.4%	46.1%	96.9%
PSP vs. Control	100%	97.7%	98.2%	91.6%	100%	81.8%	95.5%	92.7%	81.8%	95.4%
MSA vs. Control	70.0%	88.6%	85.2%	58.3%	92.9%	70.0%	88.6%	85.2%	58.3%	92.9%
PD vs. Control	61.9%	56.8%	60.7%	81.4%	32.9%	59.7%	54.5%	58.4%	80.0%	30.8%

### Correlations between apparent susceptibility and clinical scores

In the PD group, we evaluated correlations between susceptibility levels in the different regions and the clinical scores from Unified Parkinson’s Disease Rating Scale Part III (UPDRS-III) and Hoehn & Yahr ratings. For this analysis, we used Pearson partial correlation while controlling for age, gender and disease duration. There was a significant correlation between putaminal susceptibility and UPDRS-III in the PD (R = 0.213, p = 0.015). The remaining tests between susceptibility levels and UPDRS-III or Hoehn & Yahr did not show any significant correlations. We also assessed correlations between disease duration and regional susceptibility while controlling for age and gender. In these tests, we found significant correlations in the PD group in the globus pallidus (R = 0.198, p = 0.023) and in the substantia nigra (R = 0.251, p = 0.004). Plots of the statistically significant correlations are shown in Fig. 3G,I. Due to the small number of subjects with PSP and MSA, no correlation tests were carried out in these groups.

## Discussion

We performed QSM processing on standard clinical SWI sequences to create maps of apparent susceptibility and can thus demonstrate different patterns in parenchymal susceptibility in a prospective cohort of different parkinsonian disorders and healthy controls. We have processed magnitude and high-pass filtered phase data from standard clinical SWI sequences to demonstrate different patterns in parenchymal susceptibility between the different parkinsonian disorders and healthy controls. We showed a very promising performance in the separation of PSP from the other groups both using cutoffs on ROC curves and with discriminant classification analysis. The most prominent findings were seen in the red nucleus in PSP. This is in line with pathological studies reporting an abundance of tufted astrocytes as a sign of neurodegeneration in this region^15^. When comparing PSP to PD, we found a diagnostic performance with sensitivity 90.9% and specificity 97.0% in the substantia nigra, sensitivity 100% and specificity 91.0% in the red nucleus, and sensitivity 90.9% and specificity 89.6% in the dentate nucleus. The diagnostic performance was further increased in separating PSP from PD when including susceptibility levels from all studied regions in discriminant analyses. We here showed a sensitivity of 100% and specificity of 97.0% using LOOCV, which we find promising. The elevated susceptibility levels in the putamen and the dentate nucleus in MSA are coherent with known neuropathological processes with putaminal degeneration and subtypes with cerebellar involvement within this condition^16^, and thus could also be potential biomarkers. The relatively low sensitivity and specificity in separating MSA from PD could be argued to also be influenced by the fact that MSA can be a difficult clinical diagnosis, which could possibly affect the gold standard in this study^17^. We also showed correlations between UPDRS-III-scores and putaminal susceptibility in the PD group. This was consistent with the findings in an earlier study^14^ and with pathological processes in PD^1^. The correlations between disease duration in PD and susceptibility levels in globus pallidus and the substantia nigra suggests that susceptibility gradually increases with the disease progression.

The present study extended the results of a previous study^14^, where a smaller and less coherent retrospective cohort was examined. In the previous study, 62 patients with PD, 15 patients with PSP, 11 patients with MSA and 14 healthy controls underwent SWI using four slightly different sequences, across both 1.5 and 3T scanners, and results were pooled. In the current study, we were able to show that the majority of previous retrospective findings are corroborated in this larger and improved study. Here, the participants have been recruited prospectively and all were imaged on the same MRI scanner with a SWI sequence and consistent acquisition parameters, which was not the case in the earlier study. We have also used blinding of diagnosis during all segmentation processing. Considering known pathologies in PSP and cerebellar involvement in variants of MSA, we also expanded the number of investigated regions to include the cerebellar dentate nucleus as a new ROI^4,16^. The major findings of the previous study, with markedly elevated susceptibility levels in mesencephalic nuclei in PSP compared to all other groups, have been validated in this larger cohort. Reviewing literature, we found that our current results are comparable to the performance of midbrain-pons area ratio and MR Parkinsonism Index (MRPI) measurements^18^. Earlier SWI studies have described changes in susceptibility in PD and in atypical parkinsonian syndromes using visual rating of hypointensities on SWI volume, which is a combination of the magnitude and phase images of a gradient echo sequence^19,20^. A study by Haller *et al*. describes classification of PD using support vector machine learning on SWI volumes^21^. Another use of SWI in parkinsonism is for evaluating the dorsolateral nigral hyperintensity, or “swallow tail sign”, where it has been shown that loss of this sign is strongly associated with parkinsonism^9,22^. The “swallow tail sign” however, is not able to distinguish between PD and atypical parkinsonian disorders^23^. The current trend in research is to move away from the arbitrary signal intensities on standard SWI to phase-based QSM, which provides a quantitative estimate of tissue susceptibility levels by deconvoluting the tissue phase with a dipole kernel. Studies using QSM have shown that PD is associated with increased susceptibility levels in the substantia nigra compared to controls^12,13^, possibly secondary to the loss of dopaminergic neurons and dysregulation of iron homeostasis as part of the neurodegenerative process. Combining morphological analyses such as the MRPI with QSM to potentially increase the diagnostic accuracy even further is an interesting direction for future studies. Moreover, it will be important to perform comparative studies in patients with classical Richardson PSP to other subtypes of PSP and patients with corticobasal syndrome^24^. Considering that differentiating early PSP and MSA is a common clinical problem, we believe that analyzing the susceptibility patterns could be of use in these situations. Further studies on uncertain and early disease would be needed to evaluate this more in-depth.

One limitation of this study was that the SWI protocol routinely applied in clinical practice only provides high-pass filtered phase images. The filtering process removes local field inhomogeneities together with the background phase. This reduces the susceptibility contrast, which could decrease the ability to find differences between groups. We did, however, demonstrate significant and diagnostically relevant separation between the groups with this method. Considering this filtered nature of the SWI phase images, the maps reflect an apparent susceptibility rather than actual susceptibility that can be obtained from QSM based on unfiltered phase data. In contrast to other studies using QSM in PD^12,13^, we did not find a susceptibility difference in the substantia nigra between PD and controls. We believe that this might in part be due to the lower susceptibility contrast obtained from our filtered phase data. We acknowledge that the diagnostic properties for moderate and somewhat late disease stage shown in this study are not directly transferrable to early disease stages. Thus, further studies are needed in order to evaluate susceptibility mapping as an early diagnostic marker, which should include individuals with atypical parkinsonian syndromes in earlier disease stages. Longitudinal studies in parkinsonian disorders are also needed to further assess the ability of these methods to monitor disease progression or measure treatment effects in trials. It should also be mentioned that while most of the cases were not confirmed by autopsy, a long-term follow-up at a clinic focused on movement disorders was performed, which should yield an acceptably high diagnostic accuracy.

In summary, we showed different patterns of brain susceptibility between PD, PSP, MSA and healthy controls, reflecting underlying regional differences in pathological processes and possibly a brain iron accumulation. We believe that these differences in susceptibility, particularly in the mesencephalic region, could be of a clinical value for the diagnostics of parkinsonian disorders, especially when faced with the diagnostic challenges in separating PSP from other parkinsonian disorders. This would be an important and useful information in terms of clinical care of these patients with regards to optimization of medication and involvement of different health professions. Furthermore, it would be of importance to have an early and correct diagnosis when including patients in clinical trials.

A previous version of this paper has been published in manuscript form as part of the doctoral thesis of Dr. Yulia Surova^25^.

## Methods

### Ethical approval and informed consent

The study was approved by the ethics committee at Lund University and performed in accordance with the Helsinki Declaration. Written informed consent was obtained from all participants.

### Participants

Participants were prospectively recruited from the Neurology Clinic at Skåne University Hospital, Sweden, between the years 2008 and 2017, as part of the Swedish BioFINDER study (http://www.biofinder.se)^26^. Exclusion criteria consisted of age above 85 years, presence of generalized malignancy, ongoing or earlier advanced abuse of alcohol or illicit drugs, presence of Alzheimer’s disease, vascular dementia, frontotemporal lobe dementia, severe psychiatric disorders, other severe neurological disease and participation in clinical drug trial with the last 30 days. As of July 4^th^ 2017, a total of 356 individuals had been included. 67 dropped out of the study before MRI examination; 42 declined MRI examination, 23 had other or unclear diagnoses, 13 had DBS prior to study, 8 had cancer, 2 had pacemakers, 1 received a transplantation and 1 had a subdural hematoma. In total, 134 patients with PD, 11 with PSP, 10 with MSA and 44 healthy controls remained for this current study. The diagnosis of PD was established by a movement disorder specialist according to the National Institute of Neurological Disorders and Stroke (NINDS) criteria^27^. The included patients with PSP and MSA met the conditions for probable disease, as determined by a movement disorder specialist, according to their respective diagnostic criteria^28,29^. In the MSA group, seven were diagnosed with MSA of parkinsonian subtype (MSA-P) and three with cerebellar subtype (MSA-C).

### Clinical assessments

Patients and healthy controls underwent a series of clinical tests and rating scales, including motor tests with UPDRS-III and Hoehn & Yahr. To assess cognitive ability a standard battery of tests, including Mini-Mental State Examination (MMSE), were conducted by a trained physician. Presence of dementia was assessed and recorded. All tests were conducted in “on state” to ensure standardization. None of the participants exhibited signs of involuntary movements, such as dyskinesia or dystonia, during testing. The clinical tests were conducted in relatively close temporal proximity to the MRI examination (median: 1.2 months before MRI, interquartile range (IQR): 3.6 months before to 2.0 months after MRI).

### MRI protocol

Brain imaging was performed on a 3 T Siemens Skyra MRI scanner equipped with a 20-channel head coil (Siemens Medical Systems, Erlangen, Germany). High-resolution 3D SWI sequences were used to obtain magnitude and high-pass filtered phase images (repetition time 27 ms, echo time 20 ms, flip angle 15°, voxel size 0.86 × 0.86 × 1.5 mm^3^). We also acquired high-resolution 3D T1-weighted images using magnetization-prepared rapid gradient-echo (MPRAGE) (repetition time 1900 ms, echo time 2.54 ms, inversion time 900 ms, flip angle 9°, voxel size 1 × 1 × 1 mm^3^) for volumetric analysis.

### Image processing

The phase and magnitude data was processed using an in-house developed pipeline consisting of FSL (version 5.0.8, http://www.fmrib.ox.ac.uk/fsl), MATLAB (version R2015a, MathWorks, Natick, USA) and STISuite (version 2.2, Duke University, Durham, North Carolina, USA)^30,31^. These methods have been thoroughly described and illustrated in a previous study^14^. To summarize, brain masks were created through FSL-BET using the magnitude images^32^. The masks were three-dimensionally eroded to ensure that no extra-cerebral tissue was present within the mask. The masks were applied to the phase images which were unwrapped using Laplacian techniques and thereafter processed using the Variable-kernel Sophisticated Harmonic Artifact Reduction on Phase data method (V-SHARP) to remove unwanted background phase. Finally, the inverse solution from field to source was calculated using the improved sparse linear equation and least-squares method (iLSQR)^30,31,33,34^. Since the parameter in these maps is calculated from high pass filtered images, it represents an apparent susceptibility.

**Figure 4:**
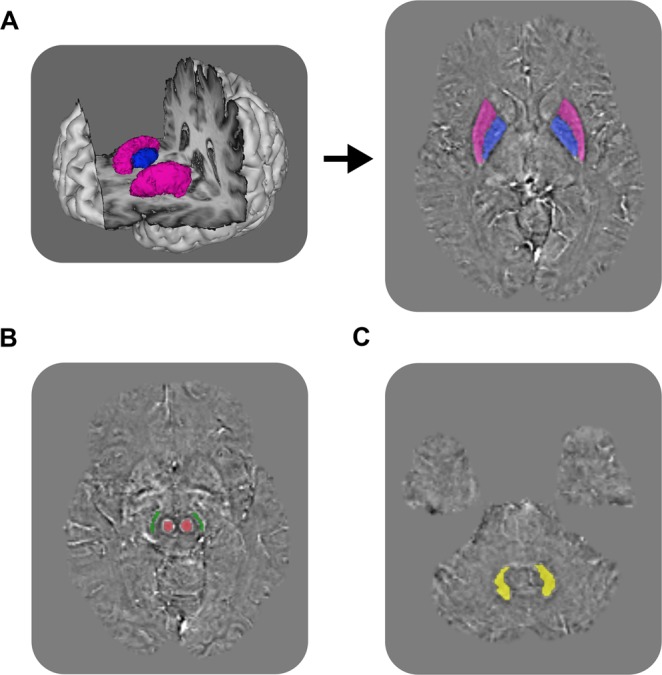
Automated segmentation of globus pallidus (blue) and putamen (magenta) on T1-weighted (**A**). Manual segmentation of substantia nigra (green), the red nucleus (red) (**B**) and the dentate nucleus (yellow) (**C**) on the susceptibility maps.

The image analysis was conducted blinded to the diagnosis and in a randomized order for all participants. Automated segmentation of the globus pallidus and putamen was performed using FSL-FIRST on the T1-weighted volumes, as illustrated in Fig. 4A^35^. The magnitude images were registered to the T1-weighted volumes and the resulting transformation applied to the susceptibility maps^36^. The masks from the automated segmentations were manually adjusted by a resident in neurology (H.S.) to ensure good fit with the susceptibility maps using ITK-SNAP (version 3.2.0, http://www.itksnap.org)^37^. The remaining ROI, consisting of the substantia nigra, the red nucleus and the cerebellar dentate nucleus, were manually delineated by a neurologist (Y.S.) using an in-house developed segmentation on the susceptibility maps, as shown in Fig. 4B,C. Apparent susceptibility from the susceptibility maps was extracted as averages of the paired structures. The selection of these particular regions was based on findings in a previous study^14^, where differences between the groups were found in globus pallidus, putamen, substantia nigra and the red nucleus. The dentate nucleus was added since PSP is known to exhibit tau pathology in this structure^4^, and the region might also be interesting in MSA considering that a subtype has prominent cerebellar involvement^16^. Additionally, ROIs were manually placed in the frontal horns of the lateral ventricles by a resident in neurology (H.S.) and all other susceptibility values were normalized to this reference region.

### Statistical analysis

Statistical analysis was performed using SPSS (version 24.0 for Mac, IBM Corp., Armonk, NY, USA). Normality was assessed using the Kolmogorov-Smirnov test. Gender distribution between the groups was assessed using Pearson’s χ^2^ test and possible differences in age by one-way ANOVA. One-way ANCOVA was used to investigate differences in susceptibility between the groups and pairwise comparisons were performed within the ANCOVA using least significant difference (LSD). Mann-Whitney U tests were performed for pairwise comparisons of susceptibility values in the dentate nucleus for MSA and PD due to non-normality. ROC AUC and discriminant analysis were used to evaluate diagnostic performance. Confidence intervals for AUC values were calculated as described by DeLong *et al*.^38^. To investigate the diagnostic accuracy further, discriminant analysis was performed between the three patient groups and the controls, using all of the five ROI. Susceptibility values from the five paired ROIs (averaged for left and right) were entered into the discriminant analysis, providing a total of five variables per individual. Prior probabilities were set to “all groups equal” to correct for unbalanced group sizes. The discriminant analyses were performed both on the entire groups and cross-validated using LOOCV. Pearson partial correlation tests were conducted to test for correlations between susceptibility levels and clinical scales, and between disease duration and susceptibility levels. The Pearson partial correlation tests were corrected for age, gender and disease duration. For the pairwise comparisons of susceptibility levels we employed a Bonferroni-corrected α-value of 0.05/30 = 0.0017 to determine statistical significance. A p-value < 0.05 was considered significant unless otherwise specified.

## Data Availability

The datasets generated during and/or analysed during the current study are available from the corresponding author on reasonable request.
